# Computational Design of Flat-Band Material

**DOI:** 10.1186/s11671-018-2464-y

**Published:** 2018-02-26

**Authors:** I. Hase, T. Yanagisawa, K. Kawashima

**Affiliations:** 1 National Institute of Advanced Industrial Science and Technology (AIST), AIST Central 2, Umezono 1-1-4, Tsukuba, Ibaraki 305-8568 Japan; 2 IMRA Material R&D Co. Ltd., Kariya, Aichi 448-0032 Japan

**Keywords:** Flat band, Ferromagnetism, Pyrochlore, Electronic structure

## Abstract

Quantum mechanics states that hopping integral between local orbitals makes the energy band dispersive. However, in some special cases, there are bands with no dispersion due to quantum interference. These bands are called as flat band. Many models having flat band have been proposed, and many interesting physical properties are predicted. However, no real compound having flat band has been found yet despite the 25 years of vigorous researches. We have found that some pyrochlore oxides have quasi-flat band just below the Fermi level by first principles calculation. Moreover, their valence bands are well described by a tight-binding model of pyrochlore lattice with isotropic nearest neighbor hopping integral. This model belongs to a class of Mielke model, whose ground state is known to be ferromagnetic with appropriate carrier doping and on-site repulsive Coulomb interaction. We have also performed a spin-polarized band calculation for the hole-doped system from first principles and found that the ground state is ferromagnetic for some doping region. Interestingly, these compounds do not include magnetic element, such as transition metal and rare-earth elements.

## Background

Electronic and magnetic properties of materials are mostly determined by their energy dispersion. For example, electronic conductivity is high when the valence/conduction band has large energy dispersion. Magnetic properties arise when the band dispersion is small. Usually, the band dispersion is determined by the character of atomic wave function. Therefore, most magnetic compound contains magnetic elements, such as transition metal elements and rare-earth elements. If we can synthesize a magnetic material containing no magnetic element, its impact is immeasurable.

In this paper, we propose such candidate of ferromagnet without containing magnetic element by using first principles calculation. The bandwidth composed of the orbital of non-magnetic atom is usually small, but in some cases, its bandwidth becomes extremely small. This narrow band is called as flat band, and if the Fermi level is just on this flat band, it is possible to take a ferromagnetic ground state. There are many studies of flat band physics, which are summarized in the review articles [1, 2].

In this paper, we briefly introduce the flat band. At first, we consider a simple tight-binding (TB) Hamiltonian ${H}_0=\varepsilon \sum \limits_i{c}_i^{+}{c}_i+\sum \limits_{i,j}{t}_{ij}{c}_i^{+}{c}_j$ (1), where *c*_*i*_ denotes the annihilation operator on *i*-site, *ε* is the on-site energy, and the hopping integral *t*_*ij*_ is finite and isotropic (= *t*) only when the site *i* and *j* are in the nearest neighbor. Quantum mechanics shows that large hopping integral gives a large energy dispersion in most cases. For example, if *H*_0_ is defined on a simple square lattice, the obtained energy dispersion is *E* (**k**) = *ε* + 2*t* (cos*k*_x_ + cos*k*_y_). The band width *W* = 8*t*, which is proportional to *t*. Since *t* is determined by the overlap of the atomic-like wave functions, if a band consists of s- or p-orbitals, it becomes a broad band. In that case, the magnetic ground state is not expected because the spin-aligned state loses a large kinetic energy.

**Fig. 1:**
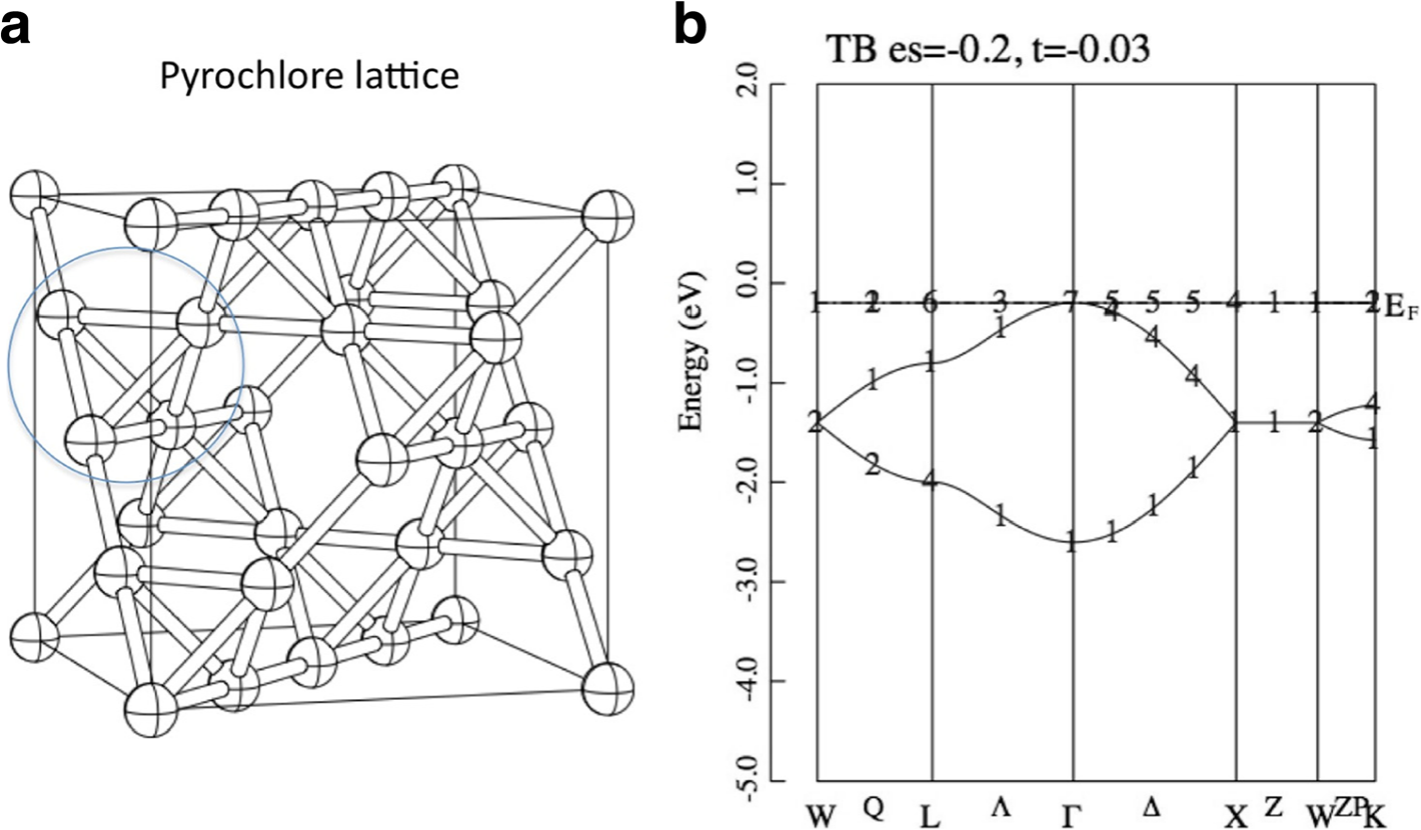
**a** Pyrochlore lattice. The balls and sticks denote the sites and bonds, respectively. This is the A-site sublattice of A_2_B_2_O_7_ pyrochlore structure. **b** Band dispersion of the tight-binding model (Eq. 1) on the pyrochlore lattice. The parameters are set as *ε* = − 0.2 and *t* = − 0.03. The unit of energy is eV. The number in (**b**) denotes the index of the irreducible representation, see ref. [34]

However, on some specific lattices, this simple relation *W* ~ *t* does not hold. For example, if *H*_0_ is defined on a pyrochlore lattice, doubly degenerated dispersionless bands appear. Pyrochlore lattice is defined as the A-site sublattice of the pyrochlore structure, see Fig. 1. We can mathematically prove the emergence of this flat band, for example, see ref [3]. There are several lattices generating flat bands besides the pyrochlore lattice, for example, 2D checker-board lattice, 2D kagome lattice, and so on [1, 2]. Interestingly, we can prove that if this flat band is half-filled, then the system has the unique ferromagnetic ground state for *any* positive value of intra-atomic Coulomb interaction *U* [4]. This type of lattice which derives flat band is known as “geometric frustrated lattice” in the word of localized spin system. In fact, a series of pyrochlore oxides R_2_Ti_2_O_7_ (R: rare-earth element) have various novel magnetic properties, such as quantum spin liquid, spin ice, and magnetic monopole [5–9]. More recently, almost perfect frustration was found in the frustrated dimer magnet Ba_2_CoSi_2_O_6_Cl_2_ [10]. An effective theory of this compound has been constructed, and this theory can explain the curious magnetic properties in the high magnetic field [11].

Besides the ferromagnetic ground state, it is theoretically suggested that flat band induces several interesting properties, such as superconductivity, quantum Hall effect, and various topological states [12–14]. Therefore, it is very important to find a compound that actually has a flat band. There are several theoretical attempts to realize the flat band using lithography [15] or photonic lattice [16]. Besides these mesoscopic materials, we note that a carefully designed 2D indium-phenylene organometallic framework (IPOF) shows an excellent flat band [17]. Interestingly, this flat band is topologically non-trivial and may serve a stage for high-temperature fractional quantum Hall effect. Despite these vigorous studies, the expected magnetic long-range order has not been achieved yet, probably because these attempts are limited for 2D system. There is another interesting study which has shown a long-range magnetic order invoked by an organic molecule absorbed on graphene [18]. However, the microscopic origin of this magnetic order is still unclear.

As is mentioned above, in order to make ferromagnetism appear using this flat band, it is necessary to adjust the Fermi level just on this flat band. In most pyrochlore oxides A_2_B_2_O_7_, the Fermi level is on the band composed of the B-site orbital. However, since the flat band has to be formed on the A-site sublattice (pyrochlore lattice), the B-site ion is to be inert. Moreover, since the hopping integral needs to be isotropic, the Fermi level must be on the s-orbital of the A-site.

Imposing the above conditions, we can choose the candidate of pyrochlore oxides to have the flat band at the top of the valence band:

$$ \left(\mathrm{a}\right)\ {{\mathrm{A}}^{1+}}_2{{\mathrm{B}}^{6+}}_2{\mathrm{O}}_7,\kern0.75em \left(\mathrm{b}\right)\ {{\mathrm{A}}^{2+}}_2{{\mathrm{B}}^{5+}}_2{\mathrm{O}}_7,\kern1em \left(\mathrm{c}\right)\ {{\mathrm{A}}^{3+}}_2{{\mathrm{B}}^{4+}}_2{\mathrm{O}}_7. $$

Since the top of the valence band has the A-s character, A-site ion is typically (a) Tl^1+^; (b) Sn^2+^, Pb^2+^; and (c) Bi^3+^. All of these ions have the (5s)^2^ or (6s)^2^ configuration. The B-site must be inert, so we can choose (a) Mo^6+^, W^6+^; (b) Nb^5+^, Ta^5+^; and (c) Ti^4+^, Sn^4+^. All of these B-site ions have closed shell, i.e., (*n*d)^0^ or (*n*p)^0^ configuration where *n* = 3, 4, 5.

Among the above combination, we focus on three compounds:

$$ \left(\mathrm{a}\right)\ {\mathrm{Tl}}_2{\mathrm{Mo}}_2{\mathrm{O}}_7,\kern0.75em \left(\mathrm{b}\right)\ {\mathrm{Sn}}_2{\mathrm{Nb}}_2{\mathrm{O}}_7,\kern0.75em \left(\mathrm{c}\right)\ {\mathrm{Bi}}_2{\mathrm{Ti}}_2{\mathrm{O}}_7. $$

The compounds (b) Sn_2_Nb_2_O_7_ and (c) Bi_2_Ti_2_O_7_ have already been synthesized [19–23], while (a) Tl_2_Mo_2_O_7_ has not been reported yet. However, an analogous pyrochlore oxide Tl_2_Ru_2_O_7_ has been already synthesized and shows unique metal-insulator transition [24]. Since the atomic radius of Mo and Ru are similar, we expect that Tl_2_Mo_2_O_7_ can be synthesized in certain condition. Interestingly, both (b) Sn_2_Nb_2_O_7_ and (c) Bi_2_Ti_2_O_7_ are known to be a candidate of photocatalytic material.

We have performed a first principles calculation for these compounds. This paper is organized as follows: In the “Methods” section, the method of calculation and the crystal structures that we calculated are described. In the “Results and Discussion” section, we show the calculated results and give some discussion. Summary is described in the “Conclusions” section.

## Methods

We have calculated the electronic structure of Tl_2_Mo_2_O_7_, Sn_2_Nb_2_O_7_ and Bi_2_Ti_2_O_7_ from first principles. For simplicity, we assumed that they all have ideal A_2_B_2_O_6_O′ pyrochlore structure. Since there are two oxygen sites, so we call them O and O′ to distinguish them. We have used a full-potential augmented plane-wave (FLAPW) scheme and the exchange-correlation potential was constructed within the general gradient approximation [25]. We used the computer program WIEN2k package [26]. The parameter *RK*_max_ is chosen as 7.0. The *k*-point mesh is taken so that the total number of mesh in the first Brillouin zone is ~ 1000. We have also optimized the crystal structure, with fixing the space group symmetry. The crystal structure of A_2_B_2_O_6_O′ is as follows: Space group Fd-3m (#227), A (0,0,0), B (1/2,1/2,1/2), O (*x*,0,0), and O′ (1/8,1/8,1/8). For Sn_2_Nb_2_O_7_ and Bi_2_Ti_2_O_7_, we used experimental lattice parameter. For Tl_2_Mo_2_O_7_, we also optimized the lattice parameter (*a*) and obtained *a* = 10.517 Å, which is very close to the recent experimental lattice parameter for the analogous compound Tl_2_Ru_2_O_7_ [27]. In this structure, the only one free parameter is the position of O (= *x*). The convergence of atomic position is judged by the force working on each atom that is less than 1.0 mRy/a.u.

## Results and Discussion

### Band Structure

**Fig. 2:**
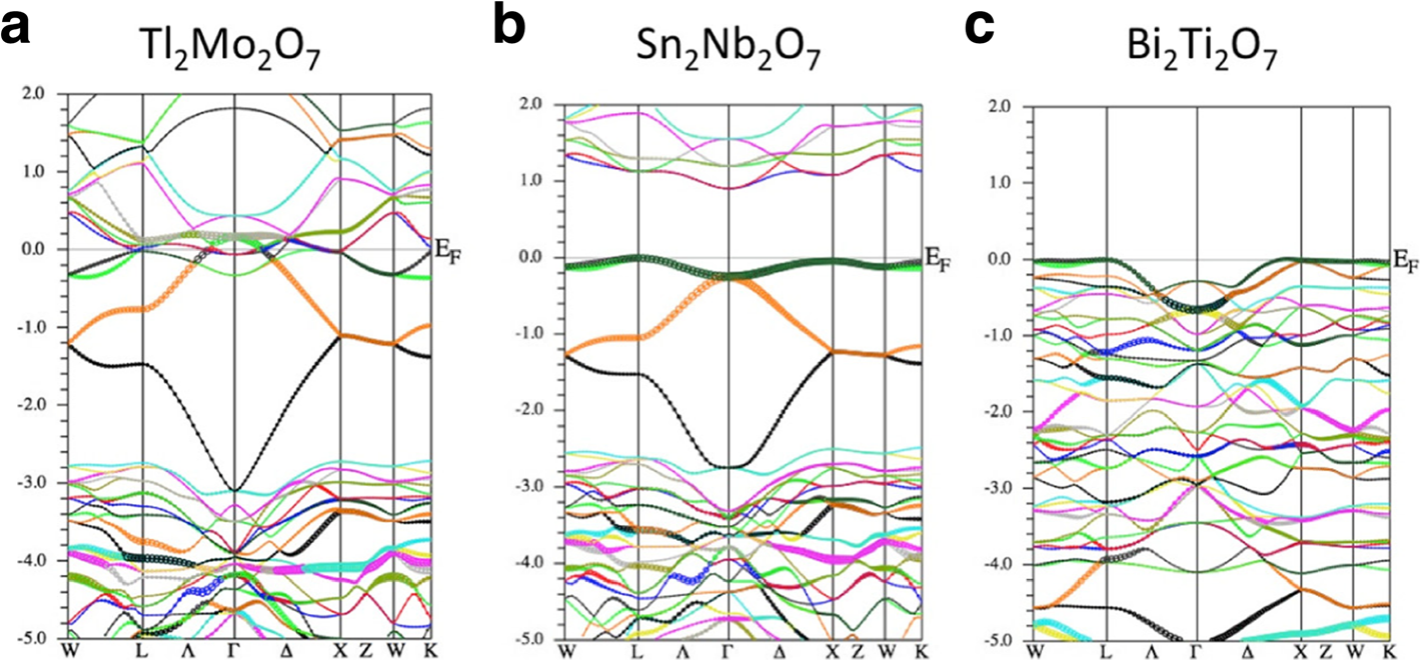
Electronic band structure of (**a**) Tl_2_Mo_2_O_7_, (**b**) Sn_2_Nb_2_O_7_, and (**c**) Bi_2_Ti_2_O_7_. The unit of energy is eV.

Figure 2 shows the energy band dispersion of Tl_2_Mo_2_O_7_, Sn_2_Nb_2_O_7_ and Bi_2_Ti_2_O_7_ from first principles. First, we focus on the middle panel, Sn_2_Nb_2_O_7_. The obtained band dispersion well agrees with the previous studies, while the existence of the quasi-flat band was not referred [19, 28]. We see that the shape of the top of the valence band (− 3~0 eV) is similar to the tight-binding model shown in Fig. 1b. This agreement is rather surprising because this model only use two parameters, *ε* and *t*. So as a first approximation, the valence band of Sn_2_Nb_2_O_7_ is described by a TB band consisting of the “Sn-s” orbitals. Here, we note that these “Sn-s” orbitals are the anti-bonding orbitals consisting of Sn-s and O′-p orbitals. The main difference between the ab-initio bands and the TB bands is the flatness of the band at the energy ~ 0 eV, which means that the hopping integrals other than the nearest neighbor Sn atoms is also needed to fit the ab-initio bands precisely.

Next, we discuss the band structure of Tl_2_Mo_2_O_7_, shown in the left panel of Fig. 2. We can see that the shape of the valence band of Tl_2_Mo_2_O_7_ is almost the same with that of Sn_2_Nb_2_O_7_, indicating the existence of the flat band in Tl_2_Mo_2_O_7_. However, the conduction band lowers its energy and the band gap is collapsed. The Mo-d band is partially occupied unlike the case of Sn_2_Nb_2_O_7_, indicating that the formal ionic configuration Tl^1+^_2_Mo^6+^_2_O^2−^ _7_ is not appropriate. This result suggests that the analysis by the point-charge model is quite effective, which suggests that A^1+^_2_B^6+^_2_O_7_ is not a stable configuration for pyrochlore oxides. The Tl-s flat band is entangled with the Mo-d band, similar to the case of an analogous pyrochlore oxide Tl_2_Ru_2_O_7_ [29]. A metal-insulator transition is found in Tl_2_Ru_2_O_7_ and its cause is ascribed to the hidden Tl-s flat band [30]. We can expect that this metal-insulator transition will also take place in Tl_2_Mo_2_O_7_ if it was synthesized.

Finally, we discuss the band structure of Bi_2_Ti_2_O_7_, shown in the right panel of Fig. 2. The obtained band dispersion well agrees with the previous study [31]. Despite the different shape of the valence band between Bi_2_Ti_2_O_7_ and Sn_2_Nb_2_O_7_, the top of the valence band of Bi_2_Ti_2_O_7_ is very flat in most part of the symmetry axis in the Brillouin zone. Since the shape of the band is different from that of Fig. 1, the origin of this partial quasi-flat band cannot simply be found in the flat band on the pyrochlore lattice. Nevertheless, the quasi-flat band and resulting high density of states (DOS) are sufficient to expect realization of ferromagnetism when doping holes. We discuss this point in the next subsection.

### Ferromagnetic States

In the previous subsection, we found a quasi-flat band at the top of the valence band in Sn_2_Nb_2_O_7_. For Bi_2_Ti_2_O_7_, we also found a partial quasi-flat band. Since they are insulator, we have to introduce holes into the quasi-flat band to induce ferromagnetism. In the case of the perfect flat band, *any* value of on-site Coulomb interaction *U* causes ferromagnetic ground state when the flat band is half-filled [4]. This means that even a well-extended atomic s- or p-orbital can cause the ferromagnetic ground state. As for the case of quasi-flat band, a numerical study shows that a certain large *U* > *U*_c_ can induce ferromagnetism, where *U*_c_ is the critical value and *U*_c_ has the order of the bandwidth *W* [32]. Since the estimation of *U* and *U*_c_ is difficult in actual compound, instead we performed a spin-polarized ab-initio calculation. Considering that the band calculation has been successful for describing the ferromagnetic ground state of bcc Fe which also has a narrow band, our approach will be justified. In order to simulate hole doping, we substitute N for O′, namely we calculated Sn_2_Nb_2_O_6_N and Bi_2_Ti_2_O_6_N. Since this substitution reduces two electrons per primitive unit cell (one electron per formula unit), the quasi-flat band becomes half-filled.

**Fig. 3:**
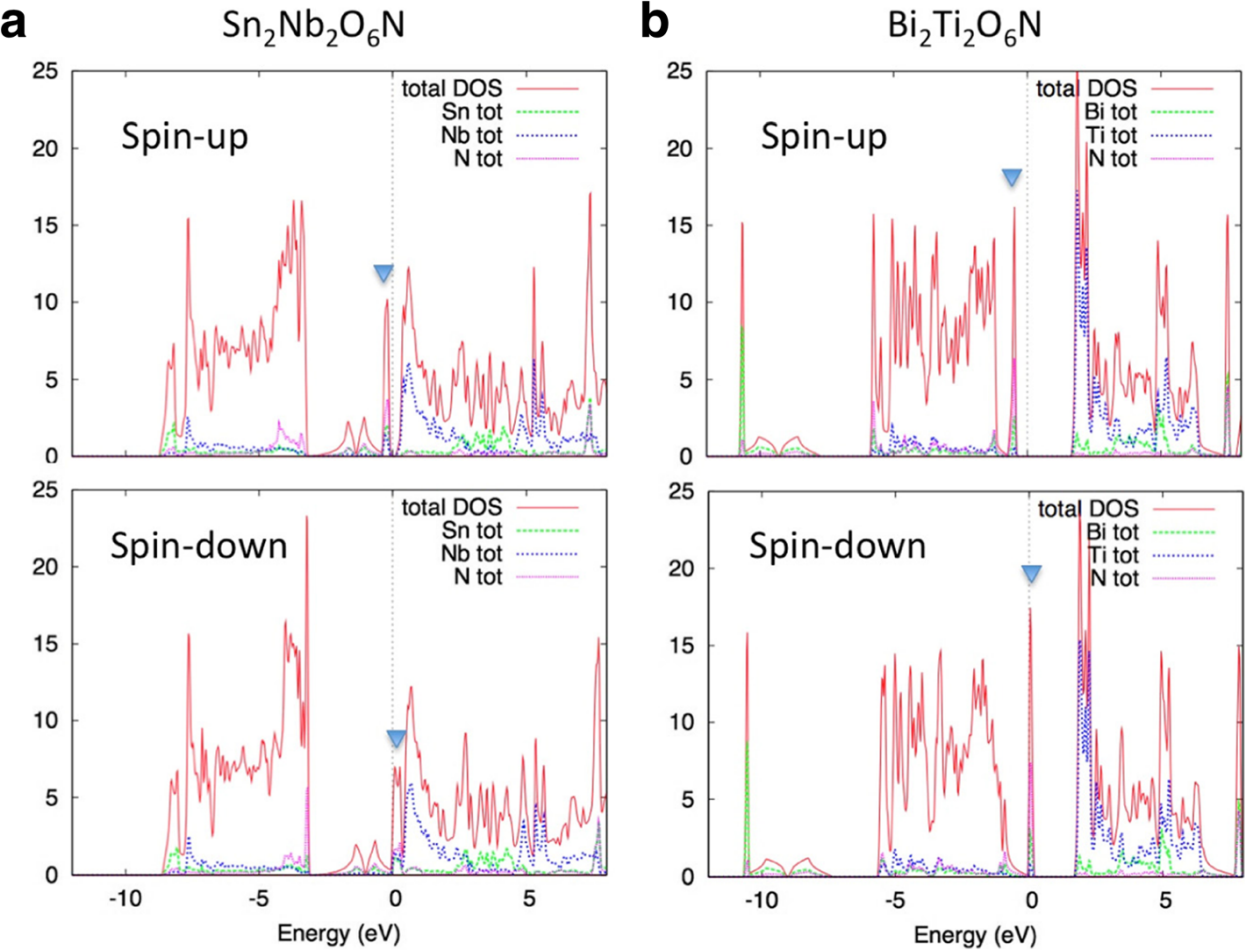
DOS curve of (**a**) Sn_2_Nb_2_O_6_N and (**b**) Bi_2_Ti_2_O_6_N for spin-polarized state. Filled triangle shows the position of the quasi-flat band

Figure 3 shows the DOS curve for Sn_2_Nb_2_O_6_N and Bi_2_Ti_2_O_6_N. The abovementioned quasi-flat band forms a sharp peak just around the Fermi level. We can see that both compounds become half-metallic, i.e., the spin state of the electron with energy *E* = *E*_F_ (Fermi energy) is fully polarized. The total magnetic moment *M* is 2.00 μ_B_ per primitive unit cell for both compounds, also indicating that the conduction electrons are fully spin-polarized. The exchange splitting between up-spin and down-spin band is ~ 0.3 eV for Sn_2_Nb_2_O_6_N and ~ 0.4 eV for Bi_2_Ti_2_O_6_N. These values are much smaller than the exchange splitting in bcc Fe, ~ 2 eV. Since the exchange splitting is approximately determined by the atomic wave function [33], the d-band has larger exchange splitting than the s- or p-band. Nevertheless, since Sn_2_Nb_2_O_6_N and Bi_2_Ti_2_O_6_N have very small bandwidth, the exchange splitting exceeds the bandwidth and half-metallic ground state realizes.

## Conclusions

In this paper, we have shown a guiding principle to design flat-band compound. According to this principle, we chose three pyrochlore oxides and investigated their electronic structure by first principles study. Combined with a tight-binding analysis, we found that some compounds actually have quasi-flat band. We also found that hole doping toward these compounds leads to the ferromagnetic ground state, despite these compounds do not contain magnetic element. These findings will be a large step to realize not only a flat-band system in a compound, but also a ferromagnet without including magnetic element.

## Abbreviations

DOS: Density of states
FLAPW: Full-potential augmented plane wave
TB: Tight-binding
